# How well do practicing radiologists interpret the results of CAD technology? A quantitative characterization

**DOI:** 10.1186/s41235-022-00375-9

**Published:** 2022-06-20

**Authors:** Fallon Branch, K. Matthew Williams, Isabella Noel Santana, Jay Hegdé

**Affiliations:** 1 Department of Neuroscience and Regenerative Medicine, Medical College of Georgia, Augusta University, Augusta University, DNRM, CA-2003, 1469 Laney Walker Blvd, Augusta, GA 30912-2697 USA; 2 Department of Psychological Sciences, Augusta University, Augusta, GA USA; 3 Department of Ophthalmology, Medical College of Georgia, Augusta University, Augusta, GA USA; 4 James and Jean Culver Vision Discovery Institute, Augusta University, Augusta, GA USA; 5 The Graduate School, Augusta University, Augusta, GA USA

**Keywords:** Assistive technologies, Base rate neglect, Base rate fallacy, Computer-assisted diagnosis, Miss rate neglect

## Abstract

Many studies have shown that using a computer-aided detection (CAD) system does not significantly improve diagnostic accuracy in radiology, possibly because radiologists fail to interpret the CAD results properly. We tested this possibility using screening mammography as an illustrative example. We carried out two experiments, one using 28 practicing radiologists, and a second one using 25 non-professional subjects. During each trial, subjects were shown the following four pieces of information necessary for evaluating the actual probability of cancer in a given unseen mammogram: the binary decision of the CAD system as to whether the mammogram was positive for cancer, the true-positive and false-positive rates of the system, and the prevalence of breast cancer in the relevant patient population. Based only on this information, the subjects had to estimate the probability that the unseen mammogram in question was positive for cancer. Additionally, the non-professional subjects also had to decide, based on the same information, whether to recall the patients for additional testing. Both groups of subjects similarly (and significantly) overestimated the cancer probability regardless of the categorical CAD decision, suggesting that this effect is not peculiar to either group. The misestimations were not fully attributable to causes well-known in other contexts, such as base rate neglect or inverse fallacy. Non-professional subjects tended to recall the patients at high rates, even when the actual probably of cancer was at or near zero. Moreover, the recall rates closely reflected the subjects’ estimations of cancer probability. Together, our results show that subjects interpret CAD system output poorly when only the probabilistic information about the underlying decision parameters is available to them. Our results also highlight the need for making the output of CAD systems more readily interpretable, and for providing training and assistance to radiologists in evaluating the output.

**Supplementary Information:**

The online version contains supplementary material available at 10.1186/s41235-022-00375-9.

## Significance

Computer-aided detection (CAD) technology was first approved for clinical use—specifically for breast cancer detection—by the US Food and Drug Administration (FDA) in 1998. Since then, the use of CAD technology has increased dramatically, especially in clinical fields in which medical images play a central role, such as radiology, pathology, neurology, and surgery (Doi, 2007; El-Baz & Suri, 2018, 2020; Fraioli et al., 2010; Mansourian et al., 2021; Regge & Halligan, 2013; Schlegl et al., 2018).

The leading sub-specialty for CAD use in radiology is screening mammography, or periodic screening of asymptomatic women for breast cancer (Helvie & Bevers, 2018; Hooshmand et al., 2021). CAD is currently used for a majority of screening mammograms in the US, and costs hundreds of millions of dollars each year (Lehman et al., 2015). By 2016, over 92% of facilities in the US that offered digital mammography used a CAD system for breast cancer detection (Keen et al., 2018). This overall trend holds worldwide, including in many developing countries (Ikeda & Miyake, 2017).

However, several studies have shown that the use of CAD does not improve diagnostic accuracy (de Hoop et al., 2010; Lehman et al., 2015; Regge & Halligan, 2013). Some studies have raised the possibility that one reason why CAD systems do not enhance detection of cancer of the colon (Regge & Halligan, 2013) and of the lung (de Hoop et al., 2010) is that radiologists fail to interpret the results of CAD systems properly. Thus, a major challenge in improving the efficacy of CAD technology in radiology is to ensure that radiologists properly interpret the results of CAD systems (Keen et al., 2018; Yanase & Triantaphyllou, 2019).

To help lay the groundwork for this endeavor, the present study focused on first understanding *how* radiologists interpret CAD information.

## Experiment 1: Interpretation of CAD system decisions by practicing radiologists

The process of interpreting CAD results is a complex one. This is in part because CAD systems typically provide multiple types of output information, including the region/s of interest (ROI/s) in a given medical image. It is also because radiologists typically evaluate the CAD output together with the relevant medical images of, and other clinical information about, the patient (Keen et al., 2018; Lehman et al., 2015; Regge & Halligan, 2013; Yanase & Triantaphyllou, 2019). Moreover, the exact nature of the output information and the guidelines for CAD use can vary depending on the particular CAD system, the clinical practice, and regulatory environments (Yanase & Triantaphyllou, 2019).

Trying to address this forbiddingly complex problem all at once would require a prohibitively large study. Therefore, for practical reasons, we adopted a layered, ‘divide and conquer’ approach, whereby we would first focus on understanding the various basic components of the process—especially the underlying cognitive mechanisms—individually, and then seek to understand the process as a whole. The two experiments described in this report represent the first part of this multilayered approach.

Given the aforementioned significance of screening mammography, we used it as the exemplar case. For the practical reasons noted above, our study did not address CAD use in mammography as a whole. Rather, it examined how radiologists interpreted the binary decision of the CAD system as to whether a mammogram was positive for cancer without seeing the mammogram itself.

Experiment 1 sought to address the following simple issue: If a CAD system examines a mammogram from a particular cohort of patients and reports only whether the mammogram was positive or negative for cancer, how do the radiologists arrive at their own estimates of the chances that the given unseen mammogram is actually positive for cancer? How well do they account for the various underlying probabilistic factors? This aspect of CAD use is admittedly removed from the actual clinical decision-making. The rationale for focusing first on this seemingly abstract cognitive process is that the actual clinical decisions must take these perceived probabilities into account, however implicitly.

## Methods

### Subjects

All procedures used in this study were approved in advance by the Institutional Review Board of Augusta University, Augusta, GA, USA. The study was carried out under the aegis of the Perception Laboratory testing facility supported by the US National Cancer Institute at the 2018 and 2019 annual meetings of the Radiological Society of North America (RSNA) in Chicago, IL, USA.

Twenty-eight practicing radiologists (six of whom were women and 22 of whom were from the US) participated in Experiment 1. Three of the subjects were practicing mammography specialists with an average of 29.3 years (median, 30 years) of experience. The remaining 25 subjects were attending radiologists who specialized in another subspecialty of radiology, and/or were trainees (residents or fellows) who had completed an average of 10.9 years (median, 5 years) of radiological experience. Since these demographic variables did not significantly affect the responses of the subjects (data not shown), we pooled the data across all 28 subjects.

The radiologists volunteered for this study and provided informed consent with the proviso that each could only spare a few minutes of participation. This practical, unavoidable constraint limited the total number of trials each radiologist could perform to 20.

### Task paradigm

Radiologists were simultaneously given four items of information on a computer screen:

i. the base rate of breast cancer in the given cohort of patients [i.e., *p*(*C*) in Eqs. 1a,b below],
ii. the hit rate *p*(*D*|*C*) of a hypothetical CAD system for breast cancer detection,
iii. the false alarm rate *p*(*D*|-*C*) of the system, and
iv. the categorical (i.e., binary) decision of the system as to whether the given mammogram was positive or negative for cancer [*D* = 1 or *D* = 0]. No mammogram was shown (see below for caveats).

The various rates and probabilities were presented both as fractions of 1 (e.g., 0.005) and as the corresponding ‘natural frequencies’ (e.g., 5 in 1000), because previous studies have shown that some subjects, including clinicians, are more comfortable with natural frequencies (Hoffrage & Gigerenzer, 1998).

Using only the above four pieces of information, the subjects had to estimate, using a mouse-driven on-screen slider, the percent chance that the hypothetical mammogram in question was positive for breast cancer.

The subjects were not shown the mammogram itself, nor provided with any other information relevant to the task. Specifically, they were provided no further information about the hypothetical CAD system or the hypothetical mammogram examined by the system. Moreover, the subjects were given no information as to what the correct probability value was or how to estimate it.

Subjects were afforded unlimited opportunity to view the on-screen information and enter their response. The theoretically expected probability of cancer *p*(*C*|*D*), i.e., the given mammogram was actually positive for cancer (*C*) given a positive finding of cancer (*D*) by the CAD system, is given by the Bayesian formula

1a $$p(C|D) \, = \, [p\left( C \right)p(D|C)]/[p\left( C \right)p\left( {D|C} \right) \, + p\left( { - C} \right)p(D|{-}C)].$$

The corresponding expected probability when the CAD system determined that the mammogram was negative for cancer is given by

1b $$p( - C|{-}D) \, = \, [p\left( { - C} \right)p( - D|{-}C)]/[p\left( C \right)p\left( {D|C} \right) \, + p\left( { - C} \right)p(D|{-}C)].$$

It is important to emphasize that the above equations were used solely for the purposes of calculating the theoretically expected probabilities during the post hoc data analyses. Subjects were made aware neither of these equations nor of the underlying math in general, nor advised of the mathematical fact that there is a unique correct answer to the problem at hand, and it is provided by these equations.

We systematically varied the values of the aforementioned four variables and studied its effect on the probabilities reported (or, synonymously, estimated) by the radiologist. Depending on the trial, the base rate *p*(*C*) had two possible values (0.005 and 0.05); the false alarm rate *p*(*D*|-*C*) had five possible values (0.05, 0.25, 0.5, 0.75, 0.95); the hit rate *p*(*D*|*C*) had two possible values (1.0 and 0.95); and the binary decision *D* of the CAD system as to the cancer status of the mammogram had two possible values (positive or negative for cancer). This resulted in 40 unique combinations of values (i.e., trial conditions), consisting of 20 conditions in which the system decided that the mammograms were positive for cancer, and 20 other conditions in which the system decided they were negative for cancer.

Given the aforementioned constraint of having to limit ourselves to 20 trials per radiologist, we tested each radiologist with only one of the aforementioned two possible values of the hit rate. Since the results did not significantly vary depending on the hit rate (not shown), we pooled the results across all subjects.

#### Rationale for the study design

We used the above experimental paradigm for three main reasons: First, it is an established paradigm for studying problems of this type. Moreover, there is a clear-cut mathematical formulation of the underlying decision problem, making it at least as principled a choice for a task paradigm as any other (see, e.g., (Marewski & Gigerenzer, 2012)). Second, this paradigm has been used to study similar problems in a diverse array of other realms, including medicine, business, and law, just to name a few (Dahlman et al., 2016; Eddy, 2005; Kahneman et al., 1982; Koehler, 1996; Mandel, 2014). This makes it easier to compare our empirical results with those from previous studies. Finally, systematically varying the underlying probabilistic parameters across trials as described above allowed us to quantitatively estimate the effect of various potential contributing factors to the errors, unlike many previous studies (for overviews, see Koehler, 1996; Mandel, 2014)).

*Rationale for the task*. The choice of the probability estimation task was guided by multiple scientific and practical considerations. As noted above, evaluating the output of a CAD system is a complex, multifaceted task (Mandel, 2014), only one facet of which is addressed by the aforementioned probability estimation task. For instance, actual FDA-approved CAD systems not only categorize a given mammogram as positive or negative for cancer, but also indicate the region/s of interest (ROI/s) in the mammogram. Moreover, under the relevant real-world clinical conditions, radiologists are not called upon to explicitly estimate probabilities; rather, they must ultimately decide whether to recall the patient for further testing (Keen et al., 2018; Lehman et al., 2015; Regge & Halligan, 2013; Yanase & Triantaphyllou, 2019).

The reasons why we limited the task in the present experiment to probability estimation were twofold: First, as alluded to above, taking on this complex, multivariate process as a whole at the outset would make the results hard to interpret, because of numerous possible confounds. On the other hand, starting by measuring the *perceived* probabilities is an eminently principled first step. Second, the anticipated practical difficulties of enrolling a sufficient number of radiologists willing to carry out a sufficient number of trials made it advisable to test the basic effect in radiological subjects before delving into a more complex study design. Thus, starting by focusing on a single, but fundamental, component of the overall process was a principled approach.

### Data analysis

Data were analyzed using scripts custom-written in the R language. Conventional tests of significance were used when appropriate, and randomization tests (Manly & Navarro Alberto, 2021) were used otherwise. Corrections for multiple comparisons were carried out using the false discovery rate methods implemented by the *R* library *stats* (Benjamini & Hochberg, 1995). Regression analyses were carried out primarily using the *R* functions *glm*() and *lm*(). Normality and homoscedasticity of the data were tested using R libraries *olsrr*, and *MASS* (data not shown), and violin plots were drawn using the R library *vioplot*. To quantitatively compare two fitted general linear models, we used the generalized estimating equation method implemented by the *R* library *geepack* (Clogg et al., 1995; Yan & Fine, 2004; Yan et al., 2013).

## Results and discussion

### Radiologists’ cancer estimates deviate widely from the theoretically expected estimates

**Fig. 1:**
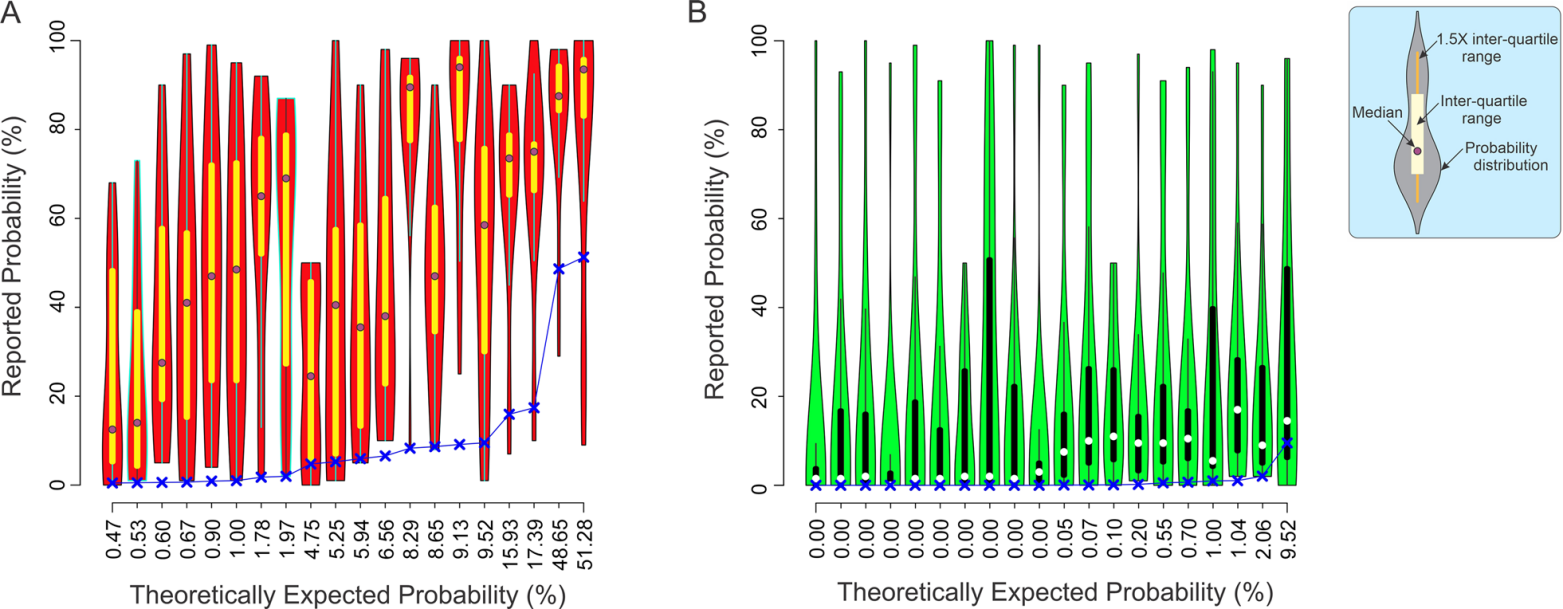
Probability estimates reported by radiologists in Experiment 1. The estimated probability of cancer (*y*-axis) in Experiment 1 plotted against the corresponding theoretically expected probabilities (*x*-axis) as standard violin plots ((Thrun et al., 2020); also see inset at *top right*). Conditions in which the CAD system determined the given mammogram to be positive or negative for cancer are plotted in (**A**, **B**), respectively. Each violin denotes one condition, i.e., one unique combination of the four probabilistic values provided to the subject. The *blue X symbols* and *line* graphically denote the theoretically expected probabilities (also see x-axis labels). See text for additional details

The radiologists’ reported (i.e., estimated) probabilities are plotted against the corresponding theoretically expected probabilities (*y* and *x axes*, respectively) as violin plots in Fig. 1. In this figure, each ‘violin’ represents the data points for a single condition, i.e*.*, a unique combination of the four aforementioned probability values provided to the subjects during a given trial.

Three aspects of the reported probabilities are especially worth noting. First, the spread of the data within each violin indicates that the reported probabilities varied considerably across trials. The maximum and minimum difference between the reported *vs*. expected percent probabilities was 100 and -42, respectively. The mean difference was 30 ± 31 (standard deviation).

Second, the reported probabilities tended to be *overestimates*, in that they were generally higher than the expected probabilities (*blue ‘X’ symbols* and *lines* in Fig. 1A, B). Indeed, this overestimation was highly significant (1-tailed *t-*test, *t* = 9.66, *df* = 557.16, *p* < 2.2^–16^). This was especially notable for conditions in which the hit rate was 1.0, and the system decided that the mammogram is negative for cancer. In such cases, it follows straightforwardly from the definition of hit rate that the probability of cancer is zero. Nonetheless, the subjects overestimated the probability in these cases as well (Fig. 1B, *first 10 violins from left*).

Third, the magnitude of the overestimation depended on the reported binary decision of the CAD system. That is, when the CAD system deemed the given mammogram positive for cancer, the radiologists’ estimated cancer probability was significantly higher than when the CAD system deemed the mammograms negative for cancer (1-tailed *t*-test, *t* = 9.6641, *df* = 557.16, *p* < 2.2 × 10^–16^; *cf.* panels A *vs.* B in Fig. 1). This suggests, on the one hand, that the radiologists did take into account the binary decision of the CAD system. On the other hand, the contribution of the binary decision to the trial-to-trial spread of the reported probabilities was not statistically significant (1-tailed *F* test, *F* (279,279) = 1.0806, *p* = 0.259). We will revisit below the influence of the binary decision of the system on the radiologists’ decisions.

### The observed effects are not fully accounted for by base rate neglect or ‘inverse fallacy’

Previous studies in other contexts have suggested that base rate neglect, where the subjects ignore the prevalence (or base rate) of an event, accounts for errors in reported probabilities (Kahneman & Tversky, 1972, 1973). If this were true in the present case, the ‘full model’ in which the expected response is calculated using all four of the independent variables (i.e., the base rate, the binary decision of the system, and the hit and false alarm rates of the system) should be statistically indistinguishable from a corresponding ‘reduced model’ in which the expected response is calculated from all independent variables *except* the base rate (Clogg et al., 1995; Yan et al., 2013). However, the two models were significantly different from each other as determined by the generalized estimating equation (GEE) modeling method for comparing models (see Methods) (GEE model, ∆ = 0.17; *p* < 0.05, corrected; data not shown), indicating that base rate neglect did not fully account for the observed results in the present case. This effect was independently confirmed using stepwise regression modeling with or without the base rate factor (data not shown).

Similarly, some previous studies have also suggested that the subjects’ reports in some comparable problem scenarios can be accounted for by the so-called inverse fallacy, whereby subjects mistake, implicitly or explicitly, the overall probability *p*(*C*|*D*) for its inverse, i.e*., p*(*D*|*C*) (for an overview, see (Koehler, 1996)). The responses expected from the inverse fallacy model were also significantly different from the responses expected from the full model (GEE model, ∆ = -0.27; *p* < 0.05, corrected), indicating that inverse fallacy did not fully account for the observed results in the present case either. Collectively, these results also suggest that conventional explanations do not adequately account for the observed effects in our case, and vindicate the approach of empirically characterizing the relevant effects.

### Relative influences of various potential contributing factors

To quantitatively characterize the factors that influenced the reported probability estimations, we regressed the reported probabilities on the four underlying independent variables (i.e., the base rate, the binary decision of the system, and the hit and false alarm rates of the system). The results showed that the base rate made a statistically insignificant contribution to the reported probability (Additional file 1: Table 1; row 2), indicating that the radiologists underweighted the prevalence of breast cancer.

On the other hand, false positive rates had a highly significant effect on the reported probabilities (row 4). The binary decision of the CAD system also had a highly significant effect (row 5). Note that these two significant effects show only that the radiologists did not neglect (or underweight) the corresponding variables, but not necessarily that the radiologists attached the precisely correct weight to these variables. This is because these effects could also arise if the radiologists attached too much weight to, i.e., overweighted, these variables.

### Radiologists overweight the false alarm rates differently depending on the binary decision of the CAD system

**Fig. 2:**
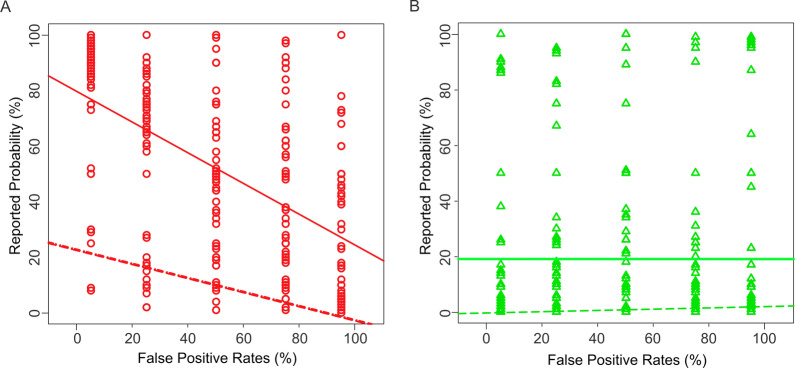
The interaction between the false alarm rate and the binary decision of the system in Experiment 1, as revealed by one-way ANCOVA. For visual clarity, the data corresponding to the two decisions of the CAD system (mammogram positive or negative for cancer) are shown separately in (**A** and** B**), respectively. In either panel, each data point denotes a single trial, and the *solid* and *dashed lines* denote best-fitting regression line for the observed and the expected responses, respectively. See text for details

To help determine if overweighting caused the aforementioned significant effects, we carried out a one-way analysis of covariance (ANCOVA; binary decision x false alarm rate). Figure 2 plots the reported probabilities (*y-axis* in either panel) as a function of the false alarm rates (*x-axis*), and of whether the CAD system deemed a given mammogram positive or negative for cancer (Fig. 2A, B, respectively). The interaction effect between the false alarm rate and the binary decision is reflected by the fact that the best-fitting regression lines (*solid lines*) have different slopes between the two panels. That is, the contribution of the false alarm rate to the reported probabilities depended on the binary decision of the CAD system (also see Additional file 1: Table 2). Thus, the subjects took into account the system’s false alarm rates when the system found the mammogram to be positive for cancer, but not if it made a negative finding.

Importantly, the radiologists’ reported probabilities were significantly higher than the expected probabilities in both panels (*dashed lines* in Fig. 2; one-tailed randomization tests; *p* < 0.05, corrected). Thus, the radiologists indeed overestimated the probabilities both when the CAD system decided that the mammogram was positive for cancer (Fig. 2A), and when the system decided the opposite (Fig. 2B).

## Experiment 2: Interpretation of CAD system decisions by non-professional subjects

As noted above, under the actual clinical conditions, radiologists using CAD system output are not called upon to estimate the probability of cancer as the subjects did in Exp. 1. Instead, radiologists must decide whether to recall the patient for further testing. For multiple reasons outlined above, Exp. 1 did not examine the latter binary decision-making process, a lacuna that the present experiment was designed to address.

In Exp. 2, non-professional subjects were given the same four items of probabilistic information as radiologists. But the subjects were required, depending on the trial block, to either estimate the cancer probability or to decide whether to recall the patient for nominal ‘further testing’. In this sense, the procedures used in Exp. 2 were a superset of those used in Exp. 1.

## Methods

### Subjects

Twenty-five non-professional subjects (19 women and 6 men; mean age, 22.8 yrs; median 23 yrs; range, 18–32 yrs) participated in this experiment. Subjects were not screened for any prior training or expertise; all persons between 18 to 65 years of age with normal or corrected-to-normal vision who volunteered for the study were enrolled.

### Task paradigms

Each subject in this experiment carried out one of two tasks, depending on the trial block. The probability estimation task (‘E’ task) was the same as the task in Exp. 1, in that the subjects were given the same four probability parameters and were asked to estimate the probability that the given mammogram was positive for cancer. There was, however, an add-on phase of the trial in this experiment that the subjects in Exp. 1 did not encounter: After the subjects estimated the cancer probability, they were presented a new screen in which they had to report how confident they were in their preceding cancer probability estimation using an on-screen slider that ranged from 0% (‘not at all confident’) to 100% (‘totally confident’). The rationale for requiring this confidence rating was that it allowed us to discern, among other things, the extent to which the subjects were confounded by the seemingly mathematical nature of the task, in which case they would tend to report low confidence on average.

The trials during the recall decision (‘D’) blocks were identical, except that instead of estimating the probability of cancer, the subjects had to make a binary decision as to whether or not to recall the patient (nominally for ‘further testing’) given the aforementioned four probabilistic parameters. After they reported their recall decision, the subjects indicated their confidence in their binary decision using an on-screen slider as above.

### Construction of trial blocks

We used the same repertoire of values as in Experiment 1, *i.e*., two possible values each for base rate (0.005 and 0.05), hit rate (1.0 and 0.95), and the decision of the CAD system (+ ve or −ve for cancer); and five possible values for false alarm rate (0.05, 0.25, 0.5, 0.75, 0.95). This resulted in 40 unique conditions.

All trials involving the E task were presented in their own block (‘E block’) of 40 randomly shuffled trials of the E type, where each trial represented one of the above 40 conditions. Similarly, the D block consisted of 40 randomly shuffled D-type trials.

Note that this design meant that the trials in the D and E blocks were mutually paired, so that for every trial in the E block that represented a given condition (i.e*.,* a given unique combination of values of the four probabilistic independent variables), there was exactly one corresponding trial in the D block, and vice versa. This allowed us to compare the given subject’s response in a given D-type trial to their response in the E-type trial, and vice versa. The rationale for separating the two sets of trials each into a block of their own (rather than intermingling them within a single block) was that the two tasks were substantially different, as noted above. Separating the trials into task-specific blocks helped simplify task instructions and minimize effects related to task switching (Kim et al., 2012).

*Testing sessions*. Prior to the start of the D blocks, we interactively explained the potential risks *vs*. benefits of the four possible outcomes of a recall decision (true and false positive, and true and false negative) to the subjects in simple, easy-to-understand language (Siu & Force, 2016). We told the subjects that the weight they attached to the various risks and benefits was entirely up to them, and that they were not required to necessarily attach the same weight to all of them.

The subjects performed each block twice, in an alternating fashion. The types of blocks were counter-rotated across subjects, so that 13 randomly selected subjects performed the blocks in an E-D-E-D block order, and the remaining 12 subjects performed them in the opposite (i.e., D-E-D-E) order. Prior to each block, subjects performed practice trials, so as to (re)familiarize themselves with the task for the given block. The data from the practice trials were discarded.

The above design meant that each subject carried out a total of 160 trials, i.e., two repetitions of each of the 40 E-type trials, and two repetitions each of the corresponding 40 D-type trials.

## Results and discussion

### Cancer estimations of non-professional subjects are comparable to those of radiologists

**Fig. 3:**
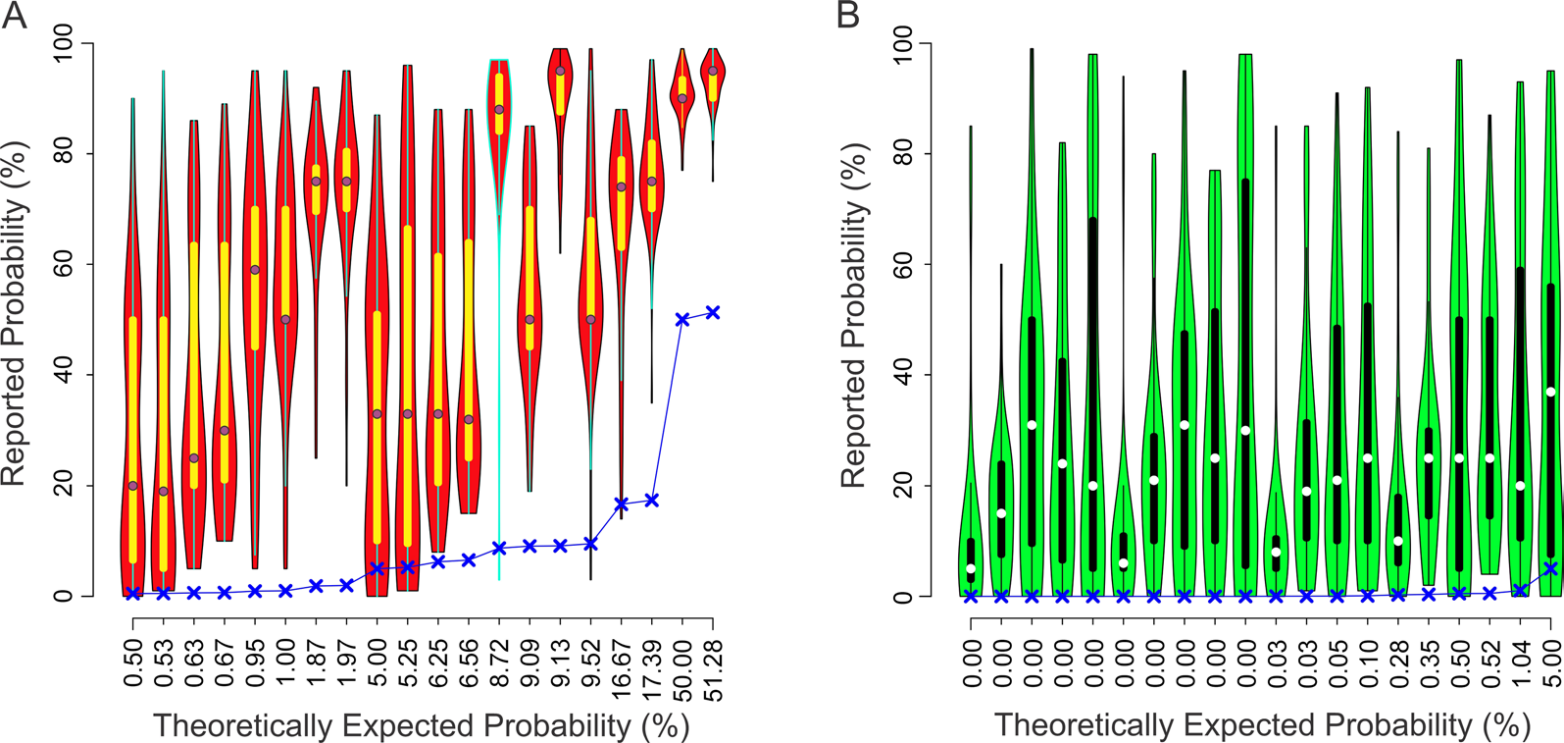
Probability estimates reported by non-professional subjects during the E-type trials in Experiment 2. The data in this figure are plotted using the same conventions as in Fig. 1. The recall rates the same set of subjects during the paired D-type trials across the same conditions are shown in the same order in Fig. 5. See text for details

The cancer probability estimations of the non-professional subjects were comparable to those of the radiologists in three important respects. First, the estimations varied widely from one trial to the next (Fig. 3A, B). The maximum and minimum difference between the reported *vs*. expected percent probabilities was 99 and -7, respectively. The mean difference was 37 ± 27.

Second, non-professional subjects also significantly overestimated cancer probability (1-tailed *t-*test, *t* = 19.11, *df* = 2037.4, *p* < 2.2^–16^). The estimates of non-professional subjects were significantly higher than those of radiologists (two-way ANOVA, subject type x conditions; *p* < 0.05 for subject type). However, the estimates were more or less uniformly higher across the board, i.e., the magnitude of increase did not vary across the conditions (*p* > 0.05 for the condition and interaction factors). This is important, because it suggests (albeit does not prove) that non-professional subjects attach similar—albeit higher—weights to the underlying factors as radiologists do.

Finally, just like the radiological subjects, the non-professional subjects tended to overestimate the probability to a greater degree when the CAD system deemed the given mammogram positive for cancer, than when the system deemed it negative for cancer (*cf. blue Xs* and* lines in* Fig. 3A* vs*. B).

One final respect in which the responses of non-professional subjects significantly differed from those of radiologists was the reaction time: The reaction times of non-professional subjects (28.25 s ± 0.71) were slightly shorter than those of radiologists (39.55 s ± 1.43; one-tailed *t*-test, *t* = 7.65, *df* = 636.64; *p* < 0.05; data not shown).

**Fig. 4:**
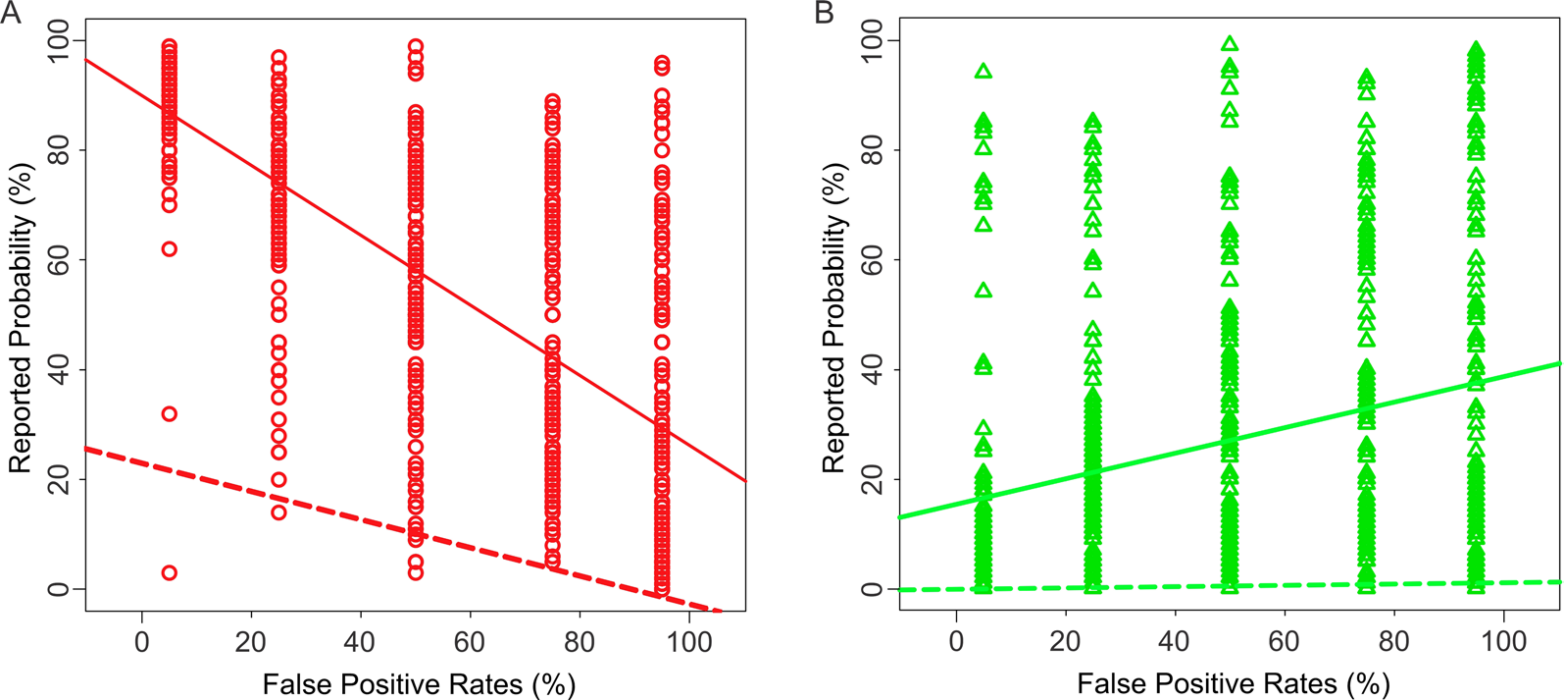
The interaction between the false alarm rate and the binary decision of the system in Experiment 2, as revealed by one-way ANCOVA (binary decision x false alarm rate). The data are plotted in this figure using the same conventions as in Fig. 2. The numeric results of the ANCOVA are shown in Additional file 1: Table 4.

Regression analysis showed that both the false alarm rates and the binary decision of the CAD system made statistically significant contributions to the subjects’ estimations, while the contribution of the hit rates was statistically insignificant (Additional file 1: Table 3). In this respect, the response patterns of non-professional subjects were similar to those of radiologists (also see Fig. 4A, B and Additional file 1: Table 4).

One significant difference was that the base rate was a significant contributor to the responses of non-professional subjects but not of radiologists (row 2 of Additional file 1: Tables 1 and 3, respectively). Recall, however, that this significant effect does not necessarily mean that the non-professional subjects correctly accounted for the variables, because these effects could also arise if the subjects attached too much weight to, i.e., overweighted, these variables.

Ultimately, the crucial similarity between the two sets of responses is that both sets of subjects significantly misestimated cancer probabilities. In this specific sense, the two sets of responses were comparable.

### Recall decisions of non-professional subjects closely reflect their cancer probability estimates

**Fig. 5:**
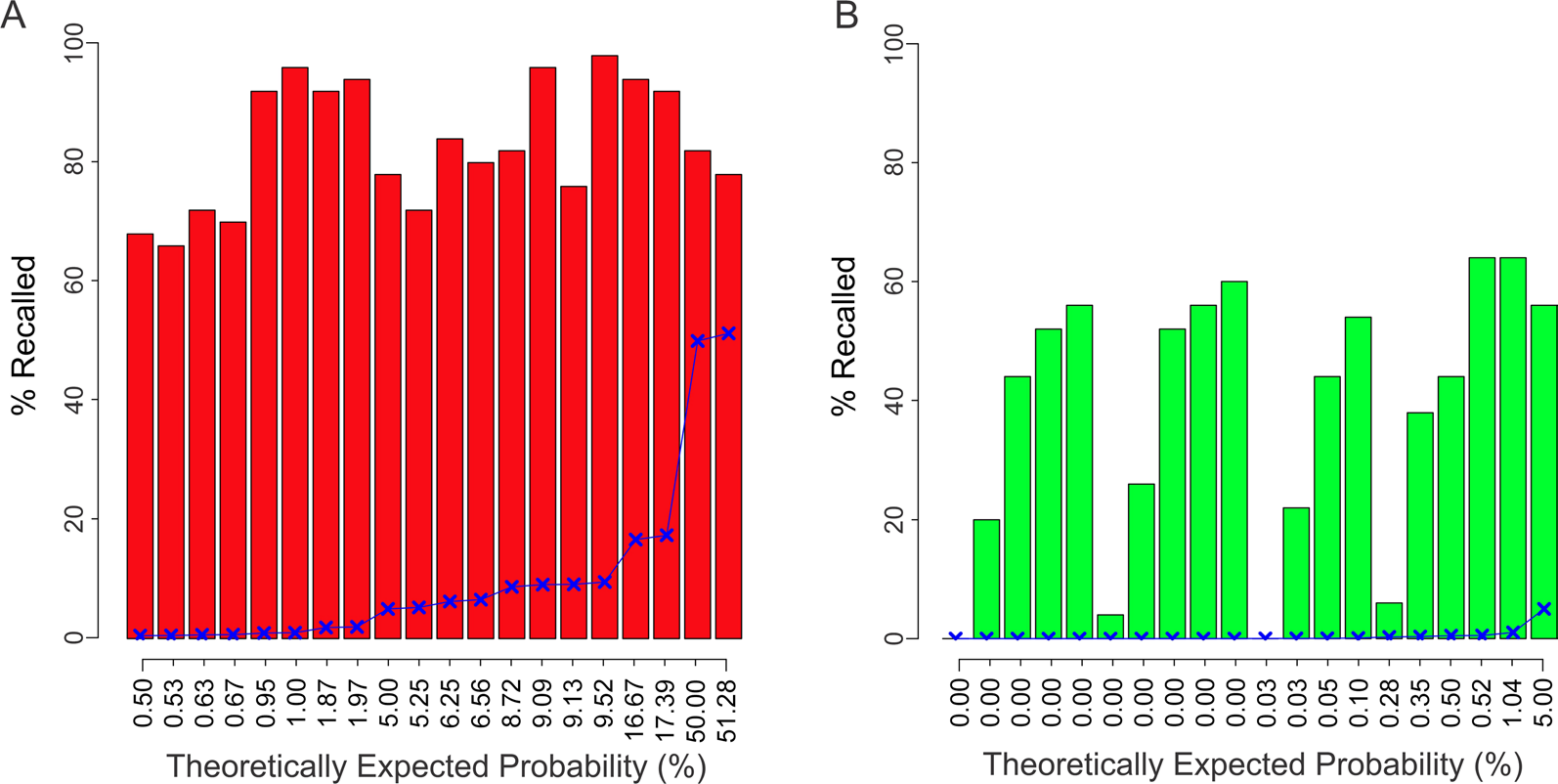
Percentage of patients recalled by non-professional subjects during D-type trials in Experiment 2. **A, B** Conditions in which the CAD system determined the given mammogram to be positive or negative for cancer (**A, B**, respectively). Note that the prominent effect of the five false alarm rate values (0.05, 0.25, 0.5, 0.75, and 0.95, in order) is especially apparent in panel B as a broadly repeating pattern of five bars starting from far left. The *blue X symbols* and *line* graphically denote the theoretically expected probabilities (also see x-axis labels). The cancer probabilities estimated by the same set of subjects during the paired E-type trials across the same conditions are shown in the same order in Fig. 3. See text for details

Across all 40 conditions, subjects decided to recall the patients for further testing an average of about 61% ± 5 of the times (Fig. 5A, B). This recall rate was significantly higher than the corresponding expected probabilities of cancer (*blue Xs* and *line*; paired *t*-test, *t* = 13.18, *df* = 39, *p* < 0.05). The subjects were much likelier to recall the patients when the system decided that the mammogram was positive for cancer than when it decided that the mammogram was negative for cancer (83% ± 2 and 38% ± 5, respectively; 2-way ANOVA, CAD decision x recall rate; CAD decision: *F* (1,36) = 53.54, *p* < 0.05; recall rate: *F* (1,36) = 14.13, *p* < 0.05).

It is possible that inadequate weighting of the underlying probabilistic factors contributed to the recall decisions. To help determine if this was the case, we carried out a logistic regression of the recall decisions (Additional file 1: Table 5). The results indicated that the false alarm rate and the decision of the system made highly significant contributions to the decision (rows 4 and 5). On the other hand, the contribution of the hit rate to the recall decisions was statistically insignificant (row 3). The base rate made a modest, but statistically significant contribution (row 2). Taken together, these findings indicate that subjects failed to adequately weight the underlying probabilistic factors in making their recall decisions.

Note that while the above results show that the recall rates were high and represented an inappropriate weighting of the underlying probabilistic factors, they do not by themselves necessarily mean that the recall rates were *unduly* high. This is because high recall rates can also arise if the subjects exercised an abundance of caution and recalled the patient when there was even the slightest chance that the mammogram was positive for cancer. However, one key observation suggests that the recalls were unreasonable at least in some cases: Subjects recalled the patients at high rates even when the probability of cancer was actually zero (see *ten left-most bars* in Fig. 5B). This allows us to reject the hypothesis that the subjects always discerned the cancer probability accurately, but simply had an extremely low threshold for recall.

To determine whether the subjects’ recall decisions were related to their cancer probability estimates, we used logistic regression to compare the recall decisions during the D-type with their paired cancer probability estimations during the E-type trials. The results showed that the cancer probability estimates could account for the recall rates in a highly significant fashion (*β* = -9.08, *z* = -3.29; *p* < 0.05, *r*^*2*^ = 0.49; also see Additional file 1: Table 6).

These results suggest (although by themselves do not prove) that the subjects’ recall decisions closely reflect their perceived probability that the given mammogram was positive for cancer. That is, the subjects were likely to have recalled the patients if they perceived (however correctly or incorrectly) that the given mammogram was positive for cancer.

### Recall decisions were not attributable to differences in confidence

Analysis of the subjects’ reported confidence ratings showed that the ratings did not differ significantly between E *vs.* D tasks (E task, 71% [mean] ± 19 [SD]; D task, 71% ± 22; paired two-tailed *t*-test, *t* = -1.02, *df* = 1999, *p* > 0.05; see Additional file 1: Figure S1).

Moreover, the reported confidence of the subjects during the D trials did not vary significantly based on whether or not the subjects decided to recall the patient (recall yes: 71% ± 21; no: 71% ± 23; two-tailed *t*-test, *t* = 0, *df* = 2422, *p* > 0.05; data not shown), indicating that the subjects’ decision to recall the patient or not was not based on how confident they felt in their decision. We obtained qualitatively similar results when we repeated this analysis using the confidence reported during the E task (recall yes: 68% ± 20; no: 74% ± 18; two-tailed *t*-test, *t* = 0, *df* = 2422, *p* > 0.05; data not shown). Together, these results indicate that the subjects felt confident about their decisions regardless of whether they decided to recall the subject; it was not as though they tended to recall the subject if they felt unsure about whether the subject had cancer. Note also that the subjects are unlikely to have expressed high confidence in their responses if they felt like they were basing their responses largely on guesswork.

Collectively, the above results show that the cancer estimation performance of non-professional subjects is directly comparable to that of the radiologists. They also show that the subjects’ recall decisions closely reflected their *perceived* likelihood of cancer (as opposed to the *actual* likelihood of cancer). This helps highlight, among other things, the fact that measuring the estimated (or perceived) likelihood of cancer is not just an exercise in scientific curiosity. Rather, it helps account for key aspects of the recall decisions.

## General discussion

Our results are significant in five main respects. First, they empirically establish that both practicing radiologists and non-professional subjects commit significant errors in estimating the actual probability of cancer based on probabilistic information. Moreover, both sets of subjects significantly *overestimate* the probability of cancer, and do so in similar ways. This indicates that the tendency to overestimate cancer probabilities is not idiosyncratic to subjects in either group.

These findings are significant in and of themselves, quite apart from what they mean for recall decisions (which we address further below). While clinicians are rarely, if ever, called upon to formally estimate probabilities, probabilistic reasoning in general does have profound clinical implications. Indeed, in his influential study that used the same probability estimation paradigm as ours, Eddy (1982) argued that “errors [in probabilistic reasoning] threaten the quality of medical care”, and that “the power of formal probabilistic reasoning provides great opportunities for improving the quality and effectiveness of medical care”. Many subsequent studies of CAD use have echoed this sentiment (de Hoop et al., 2010; Keen et al., 2018; Lehman et al., 2015; Regge & Halligan, 2013; Yanase & Triantaphyllou, 2019).

Second, and even more importantly, we show that probability estimations closely reflect the recall decisions, at least in the case of non-professional subjects. This is not necessarily to say that the subjects explicitly base their recall decisions on their estimations of cancer probability; it is possible that both cognitive processes reflect a third, unknown process.

Note, parenthetically, that our study design made it possible to reject, at least for non-professional subjects, the hypothesis that recall decisions always reflect a reasonable, if hypervigilant, strategy. This is because the subjects recalled patients even when the actual probability of cancer was literally zero.

Our study also highlights the potential usefulness of studying selected aspects of the clinical decision-making process in non-professional subjects. For instance, our results make it reasonable to hypothesize that the close relationship between probability estimations *vs*. recall decisions we demonstrate in non-professional subjects also holds for practicing radiologists. The design of Exp. 2 provides a straightforward template for testing this hypothesis.

Third, our results reveal three different, significant sources of the estimation errors by radiologists: (1) the neglect of the prevalence of breast cancer, (2) overweighting of the binary decision of the CAD system in each individual case (i.e., ‘individuating’ information), and (3) the binary decision-dependent neglect of the false alarm rate. Note that the latter two factors pertain to the CAD system per se (as opposed to the base rate, which is not a property of the CAD system). Somewhat surprisingly, non-professional subjects were better at accounting for the base rate, although their overestimations were significantly worse. The reasons for these differences remain to be established.

Importantly, our results empirically demonstrate that observed estimation errors were not fully attributable to well-known effects such as base rate neglect or overweighting of the individuating information by either group of subjects. That is, while these effects did contribute to the observed cancer probability estimates, they did not fully account for them. Incidentally, this finding highlights the importance of having empirically measured these errors in the specific context of interpreting CAD results, because our results could not have been predicted by simply extrapolating from the previous studies of the underlying estimation problem in other contexts (for a review, see (Mandel, 2014)).

Fourth, our study identifies a novel effect that significantly contributes to the estimation errors by both radiologists and non-professional subjects, namely the conditional neglect of false alarm rates. The neglect of the false alarm rate has been previously reported in the context of legal decision-making (Dahlman et al., 2016). However, to our knowledge, the present study represents the first report of a *conditional* neglect of the false alarm rate in any decision-making context.

This effect is intriguing, because it means that subjects take the system’s false alarm rates into account if the system decides that the given mammogram is positive for cancer, and neglect the false alarm rates otherwise. One possible explanation of this is that if the CAD system reports that the given mammogram is positive for cancer, then the subjects consider the false alarm rate in order to help them determine whether or not the given positive report is a *false* positive (or false alarm). On the other hand, if the CAD system determines that the given mammogram is negative for cancer, the subjects ignore the false alarm rate, presumably because the false *positive* rate is moot when the system’s finding is *negative* to begin with. There is a certain intuitive logic to this.

Note, incidentally, that the fact that the subjects do take false alarm rates into account does not necessarily mean that the subjects attach the correct weight to the rates. Indeed, our results indicate that the radiologists do not attach proper weight to this factor (i.e., they overweight it). Further studies are needed to fully characterize this effect.

Finally, another significance of our study is that we quantitatively estimate the effect of each of the aforementioned contributing factors in both estimation and recall tasks. These estimates also confirm that CAD estimation errors reflect a unique weighted combination of the underlying contributing factors. This also suggests that there is no unifying explanation that a priori accounts for all such phenomena. The closest one can come to a unifying framework of explanation is that there is a finite set of potential contributing factors that potentially apply to all problems of this type, but the observed results in any given problem scenario depend on the relative weights of the various factors in that problem scenario. Since there is no known way of predicting these weights a priori, one must empirically estimate these weights (i.e., the relative contributions) of the various factors in each case. Again, this vindicates our empirical approach.

A corollary of this is that it can be misleading and counterproductive to attribute such errors a priori to a well-known cause, such as base rate neglect, or overweighting of the individuating information.

### Probability estimation errors in other contexts

Neglect of the base rate and the overweighting of the individuating information have been shown to cause estimation errors in other contexts (Fischhoff & Bar-Hillel, 1984; Kahneman & Tversky, 1973; Mandel, 2014). Our results empirically demonstrate these effects in the context of CAD result interpretation. The neglect of the false alarm rate has been previously reported in the context of legal decision-making (Dahlman et al., 2016). However, to our knowledge, the present study represents the first report of a *conditional* neglect of the false alarm rate in any decision-making context.

Some previous studies in other contexts have shown that other approaches, such as appropriately modifying the statement of the problem and decision-work flow, can also reduce estimation errors (Hoffrage & Gigerenzer, 2004; Mandel, 2014; Wood, 1999). Our preliminary study did not address such admittedly important complexities because it simply aimed to empirically establish the existence of the aforementioned estimation and decision errors and their sources, and not how to reduce the errors.

### Additional caveats and future directions

In addition to the various caveats specific to our study noted in context throughout this report, it is worth noting one general caveat that applies to our study and more broadly to all laboratory studies of clinical phenomena: Such studies simplify, out of both practical and scientific necessity, the underlying clinical problems. We have noted throughout this report the various ways in which our study does this. However, it is worth noting, without glossing over the limitations of our study, that our results would have been essentially uninterpretable if we had not simplified the study design by removing these potential confounding variables. For instance, if we had presented the actual mammograms to the subjects, we would not have been able to ascertain the contribution of the aforementioned four pieces of probabilistic information about the CAD system to the subjects’ responses, because of the potentially confounding contributions of the mammogram and how the subjects perceived it. In removing these confounds and presenting the results along with the applicable caveats as we do, our study adopted this standard tradeoff: Seek to clarify by simplifying.

Much future work is needed to address the many questions raised by our study. These include, but are not limited to, ascertaining whether and to what extent our results, especially from non-professional subjects, are generalizable to actual clinical conditions.

An important practical implication of our results is that that training subjects to properly interpret the output of CAD systems may help improve the efficacy of CAD systems in mammography (Yanase & Triantaphyllou, 2019). It may also help if the systems explicitly provide an additional piece of information, namely the expected probability of cancer for each given mammogram.

## Supplementary Information

**Additional file 1**. Additional results from Experiments 1 and 2.

## Data Availability

The images used in the study and the deidentified data are available for non-commercial use upon reasonable request.
