# Supplementary material for: How well do practicing radiologists interpret the results of CAD technology? A quantitative characterization
Source: Cogn Res Princ Implic. 2022 Jun 20;7:52. doi: 10.1186/s41235-022-00375-9 (PMC9209598; doi:10.1186/s41235-022-00375-9)
Supplement: Supplementary file 1 — Additional file 1. Additional results from Experiments 1 and 2. [file 41235_2022_375_MOESM1_ESM.docx]

**SUPPLEMENTARY INFORMATION**

**Additional results from Experiments 1 and 2**

**How Well Do Practicing Radiologists Interpret the Results of CAD Technology? A Quantitative Characterization**

Fallon Branch^1§^, K. Matthew Williams^2§^, Isabella Noel Santana^1^, and Jay Hegdé^1,3,4,5^*

^1^Department of Neuroscience and Regenerative Medicine, Medical College of Georgia, Augusta University, Augusta, GA, USA

^2^Department of Psychological Sciences, Augusta University, Augusta, GA, USA

^3^Department of Ophthalmology, Medical College of Georgia, Augusta University, Augusta, GA, USA

^4^James and Jean Culver Vision Discovery Institute, Augusta University, Augusta, GA, USA

^5^The Graduate School, Augusta University, Augusta, GA, USA

§ These two authors contributed equally to this study.

*Corresponding Author. Email: [jhegde@augusta.edu](mailto:jhegde@augusta.edu)

* Corresponding Author: Augusta University, DNRM, CA-2003, 1469 Laney Walker Blvd, Augusta, GA 30912-2697. Email: jhegde@augusta.edu; Phone: +1-706-721-5129

Table 1. Summary of general linear modeling results in Experiment 1.

| Row # | **Independent Variable** | **Estimated Coefficient** | **Standard Error** | ***t* value** | ***p* value** |
| --- | --- | --- | --- | --- | --- |
| 1 | Null model (intercept only) | 0.50 | 0.24 | 2.14 | 0.03 |
| 2 | Base rate of cancer in the cohort | 0.80 | 0.55 | 1.47 | 0.14 |
| 3 | Hit rate of the system | -0.20 | 0.25 | -0.80 | 0.42 |
| 4 | False alarm rate of the system | -0.28 | 3.77 x 10^-2^ | -7.36 | 6.82 x 10^-13^ |
| 5 | Decision of the system about the given mammogram | 0.33. | 2.46 x 10^-2^ | 13.45 | <2 x 10^-16^ |
| 6 | Reaction time | -2.27 x 10^-7^ | 3.65 x 10^-7^ | -0.62 | 0.53 |

Table 2. Binary decision-dependent effects of false alarm rates on probability estimation in Experiment 1: Summary of ANCOVA modeling results.

| Row # | **Independent Variable** | ***df*** | **Sum of Squares** | ***F* value** | ***p* value** |
| --- | --- | --- | --- | --- | --- |
| 1 | False alarm rate of the system | 1 | 4.53 | 59.44 | 5.85 x 10^-14^ |
| 2 | Decision of the CAD system about the given mammogram | 1 | 15.20 | 199.29 | <2 x 10^-16^ |
| 3 | Interaction between the false alarm rate and the decision of the system | 1 | 4.53 | 59.35 | 6.09 x 10^-14^ |
| 4 | Residuals | 556 | 42.42 |  |  |

Table 3. Experiment 2: Summary of general linear modeling of probabilities estimated during E-type trials

| Row # | **Independent Variable** | **Estimated Coefficient** | **Standard Error** | ***t* value** | ***p* value** |
| --- | --- | --- | --- | --- | --- |
| 1 | Null model (intercept only) | 0.21 | 0.23 | 0.92 | 0.36 |
| 2 | Base rate of cancer in the cohort | 0.70 | 0.26 | 2.70 | 0.007 |
| 3 | Hit rate of the system | 0.13 | 0.23 | 0.56 | 0.58 |
| 4 | False alarm rate of the system | -0.20 | 0.02 | -11.30 | <2 x 10^-16^ |
| 5 | Decision of the system about the given mammogram | 0.31 | 0.01 | 26.82 | <2 x 10^-16^ |
| 6 | Reaction time | 5.43 x 10^-7^ | 3.47 x 10^-7^ | 1.56 | 0.12 |

Table 4. Binary decision-dependent effects of false alarm rates on probability estimation during E-type trials in Experiment 2: Summary of ANCOVA modeling results.

| Row # | **Independent Variable** | ***df*** | **Sum of Squares** | ***F* value** | ***p* value** |
| --- | --- | --- | --- | --- | --- |
| 1 | False alarm rate of the system | 1 | 8.84 | 178.9 | <2 x 10^-16^ |
| 2 | Decision of the CAD system about the given mammogram | 1 | 49.61 | 1004.5 | <2 x 10^-16^ |
| 3 | Interaction between the false alarm rate and the decision of the system | 1 | 40.95 | 829.2 | <2 x 10^-16^ |
| 4 | Residuals | 2036 | 100.55 |  |  |

Table 5. Experiment 2: Summary of general linear modeling of recall decisions during D-type trials

| Row # | **Independent Variable** | **Estimated Coefficient** | **Standard Error** | ***z* value** | ***p* value** |
| --- | --- | --- | --- | --- | --- |
| 1 | Null model (intercept only) | 1.43 | 2.03 | 0.70 | 0.48 |
| 2 | Base rate of cancer in the cohort | 8.40 | 2.32 | 3.62 | 0.0003 |
| 3 | Hit rate of the system | -2.70 | 2.08 | -1.30 | 0.19 |
| 4 | False alarm rate of the system | 1.21 | 0.16 | 7.41 | 1.3 x 10^-13^ |
| 5 | Decision of the system about the given mammogram | 2.16 | 0.11 | 19.79 | <2 x 10^-16^ |
| 6 | Reaction time | -1.18 x 10^-5^ | 5.40 x 10^-6^ | -2.19 | 0.03 |

Table 6. Experiment 2: Summary of logistic regression modeling of recall decisions during D-type trials

| Row # | **Independent Variable** | **Estimated Coefficient** | **Standard Error** | ***z* value** | ***p* value** |
| --- | --- | --- | --- | --- | --- |
| 1 | Null model (intercept only) | 5.47 | 1.63 | 3.36 | 0.001 |
| 2 | Probabilities estimated in paired E-type trials | -9.08 | 2.76 | -3.29 | 0.001 |


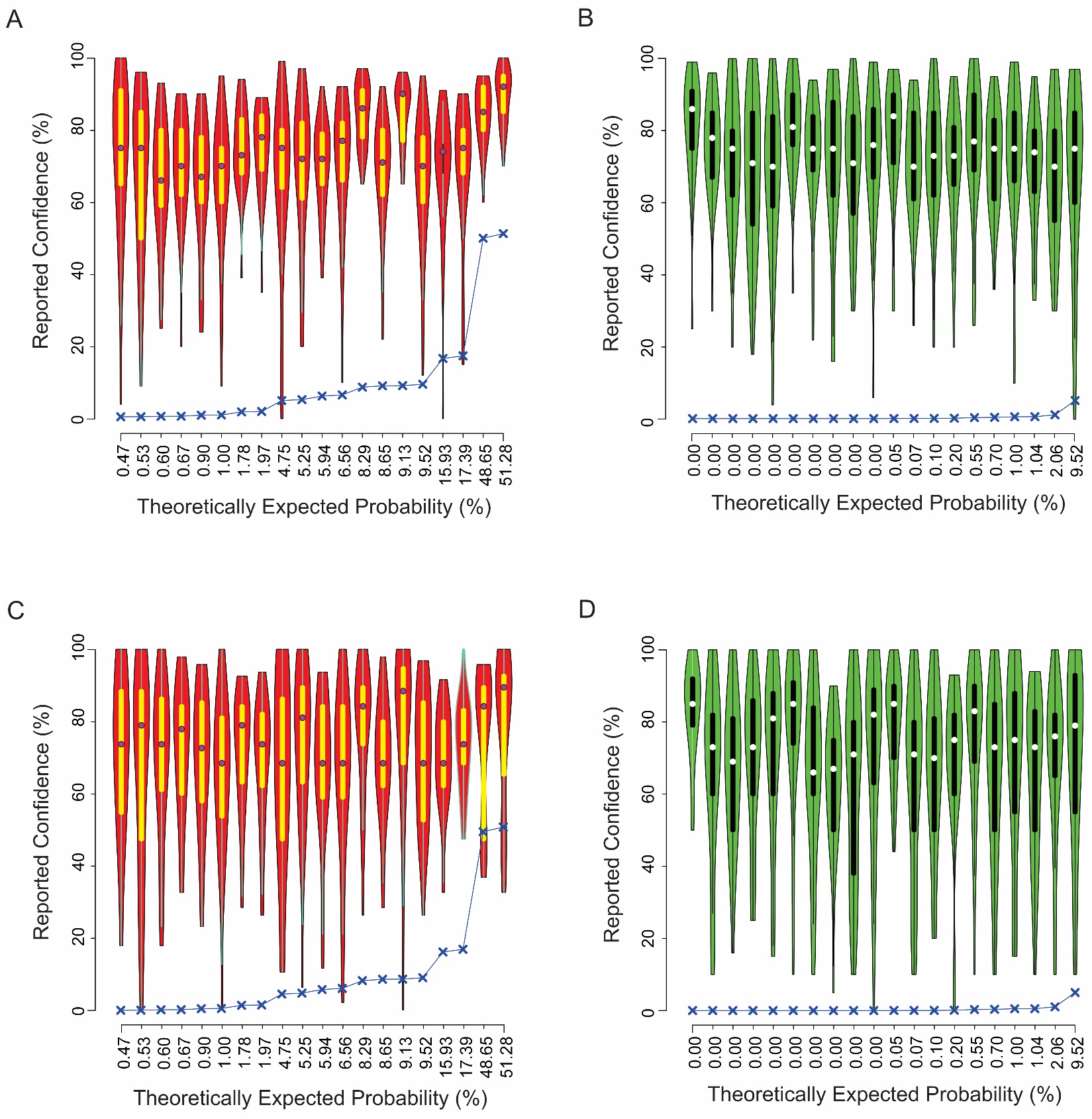


Supplemental Figure S1. Violin plots of confidence ratings of the subjects in Experiment 2. (A, B) Confidence ratings during the E-type trials. (C,D) Confidence ratings during the D-type trials. Panels A and C show the ratings during the conditions in which the CAD system deemed the mammograms positive for cancer. Panels B and D show the ratings during the conditions in which the system deemed mammograms negative for cancer. See text for details.
